# PADI2 Is Significantly Associated with Rheumatoid Arthritis

**DOI:** 10.1371/journal.pone.0081259

**Published:** 2013-12-05

**Authors:** Xiaotian Chang, Yifang Xia, Jihong Pan, Qingsong Meng, Yan Zhao, Xinfeng Yan

**Affiliations:** 1 Medical Research Center of Shandong Provincial Qianfoshan Hospital, Shandong University, Jinan, Shandong, P. R. China; 2 Research Center for Medicinal Biotechnology, Shandong Academy of Medical Sciences, Jinan, Shandong, P. R. China; Center for Rheumatic Diseases, India

## Abstract

Citrullination, a posttranslational modification of peptidyl arginine to citrulline, plays an essential role in rheumatoid arthritis (RA). Citrullination is catalyzed by a group of peptidylarginine deiminases (PADs) including PADI 1, 2, 3, 4 and 6. Many studies have indicated that the gene encoding PADI4 is a factor in susceptibility to RA. Some studies have detected PADI2 expression in RA synovial tissues, suggesting that PADI2 also plays an important role in the disease. This study evaluated the possible association between the PADI2-encoding gene and RA. Seventeen tag SNPs across the PAD locus were genotyped using a custom-designed Illumina 96-SNP VeraCode microarray. Peripheral blood samples were collected from patients with RA (n = 267), ankylosing spondylitis (AS, n = 51) and healthy controls (n = 160). The results of genotyping were verified using Sequenom MassARRAY in an independent cohort of 307 patients with RA, 324 patients with AS and 509 healthy controls. A western blot analysis was performed using synovial tissue from patients with RA (n = 7), osteoarthritis (OA, n = 7) and AS (n = 5) to determine the levels of expression of PADI2. A microarray analysis revealed a significant association between three selected PADI2 SNPs (rs2235926, rs2057094, rs2076616) and the presence of RA. The increased susceptibility to RA associated with rs2235926 (OR = 1.706733, 95% CI = [1.576366–1.866587], p = 0.000839) and rs2057094 (OR = 1.360432, 95% CI = [1.065483–1.869482], p = 0.003291) was further confirmed by the Sequenom MassARRAY. No tag SNPs in the PADI2 locus showed a significant association with AS. Increased expression of PADI2 was detected in RA synovial tissues compared with samples from patients with OA and AS. PADI2 is significantly associated with RA and may be involved in the pathogenesis of the disease.

## Introduction

Citrullination is a posttranslational modification involving the conversion of arginine residues into the amino acid citrulline. This modification affects physiology either by directly modulating protein function or by impacting immune recognition of self proteins. Rheumatoid arthritis (RA) is an autoimmune disease, and serum from RA patients contains a spectrum of autoantibodies including rheumatoid factor, anti-filaggrin autoantibody, anti-keratin antibody (AKA), anti-perinuclear factor, anti-vimentin and anti-cyclic citrullinated peptide antibody (anti-CCP) [1]–[5]. The B-cell epitopes of most RA autoantibodies contain citrulline. Antibodies directed against citrulline-containing proteins are highly specific for RA and can be detected in up to 80% of RA patients. Thus, citrullination plays an essential role in the autoimmune basis of RA [6].

Citrullination is catalyzed by a group of calcium-dependent peptidylarginine deiminase (PAD) enzymes. Five mammalian PAD family members (PAD or PADI 1–4 and 6), all encoded by a cluster of genes on chromosome 1p36, have been described and show tissue-specific distribution in most body tissues. Over the past decade, PAD and protein citrullination have been commonly implicated as abnormal pathological features in the inflammatory response. The majority of our knowledge regarding the disease-related mechanisms of uncontrolled citrullination and anti-citrullinated protein antibody (ACPA) development in RA is focused on PADI4. Recent studies have indicated that polymorphisms of the PADI4 gene confer susceptibility to RA in people of East Asian descent. Case-control association studies and mRNA stability assays indicate a strong association between the PADI4 gene and RA in the Japanese, Korean and Chinese populations. However, studies in European populations have produced conflicting results. A weak association or no association was found in Caucasian populations, including Spanish, Tunisian, British, white Hungarian, French, German, North American and Swedish populations [7]–[26]. Moreover, many studies failed to find any link between the PADI4 genotype and the presence of anti-CCP antibodies, rheumatoid factor or erosions in people with RA [19], [23], [27]–[29].

To investigate how the PAD gene and its expression are involved in the RA pathogenic process, some studies have investigated the expression and activity of other PAD isotypes in the peripheral blood and synovial fluid cells of patients with RA. Vossenaar *et* *al.* investigated the expression and activity of four isotypes of PAD in the peripheral blood and synovial fluid cells of patients with RA. They detected that transcription of PADI2 and PADI4 mRNA predominated in peripheral blood monocytes. However, PADI4 mRNA was not detectable in the macrophages that were abundant in the inflamed RA synovium. They also found that PADI2 expression was closely linked with inflammation in RA synovial tissue and that PADI2 and citrullinated proteins were present in the synovial fluid of RA patients [30]. Vossenaar *et* *al.* also examined PAD expression in synovial tissues from mice with collagen-induced arthritis and mice with streptococcal cell wall-induced arthritis. In both mouse models, PADI2 mRNA was present in the synovium but was not translated into PADI2 protein. In contrast, PADI4 mRNA, although absent from healthy synovium, was readily transcribed and translated by polymorphonuclear neutrophils infiltrating the synovial tissue during inflammation [31]. We also detected PADI4 expression in RA synovial membrane and synovial fluid in previous studies [32], [33]. The above results demonstrate that PADI2 and PADI4 are the most likely candidates among the PAD isotypes for the citrullination of synovial proteins in RA. Although, among the four isoforms of PAD, only PADI4 has been identified as an RA-susceptibility gene, it is possible that PADI2 and other PAD members are also genetic factors for RA and are involved in the pathogenic process.

Our study aimed to investigate the association between potential single nucleotide polymorphisms (SNPs) in the entire PAD locus (including the PADI1, PADI2, PADI3, PADI4 and PADI6 genes) and RA risk. We also investigated PADI2 expression in RA synovial tissues. Samples collected from patients with ankylosing spondylitis (AS) or osteoarthritis (OA) were used as controls.

## Results

### Genotyping SNPs Located in the PAD Locus

Seventeen SNPs across the gene were genotyped using a custom-designed Illumina microarray. All of the SNPs yielded genotypic data, and the study sample success rate was 98.1%. All of the SNPs except rs6695849 were found to be in Hardy-Weinberg equilibrium (p>0.05) within the healthy samples. The differences in allele and genotype frequencies between the cases and controls were compared. The frequencies of the rs2235926, rs2057094, rs2076616 and rs11203368 alleles showed a significant association with RA (p = 0.012818, 0.016166, 0.045123 and 0.000229, respectively). The genotype frequencies of the four SNPs were also significantly associated with the disease (p = 0.028584, 0.037656, 0.043905 and 0.00063, respectively). Following the Bonferroni correction, the SNPs rs2235926, rs2057094 and rs11203368 still had a significant association with RA with regards to allele frequency and genotype frequency, indicating that these three SNPs in the PADI2 and PADI4 encoding genes are significantly associated with the disease. We also investigated the potential association between PAD polymorphisms and AS. The Illumina microarray detected a significant association between rs1635564, rs1204898 and rs10788668 and AS with regards to allele frequencies (p = 0.007332, 0.016945 and 0.042589, respectively). The genotype frequency of rs1635564 also demonstrated a significant association with AS (p = 0.032555). Following the Bonferroni correction, the SNP rs1635564 in the PADI4 region still had a significant association with AS with regards to allele frequency and genotype frequency. The Illumina microarray results are shown in **Table 1** and **Table 2**. The microarray results were submitted to the NCBI Gene Expression Omnibus (GEO), and the record was approved and assigned GEO accession number GSE39428.

**Table 1:** Allele and genotype frequencies in a case control (n = 163) cohort of patients with RA (n = 266) by the illumina microassay.

Genes	dbSNP	Chrlocation	Alllele/genetype	No. of patients with RA (%)	No. of controls (%)	Fisher’s p value	Odds Ratio (%95 CI)	Case HWE (df = 1)	Control HWE (df = 1)
PADI2	rs2235926	17395281	A	245 (0.496)	125 (0.406)	0.012818	1.440482 (1.080226∼1.920884)		
			G	249 (0.504)	183 (0.594)				
			AA	55 (0.223)	20 (0.130)	0.028584		0.143098	0.07304
			AG	135 (0.547)	85 (0.552)				
			GG	57 (0.231)	49 (0.318)				
PADI2	rs2057094	17405949	A	250 (0.470)	125 (0.386)	0.016166	1.411348 (1.065483∼1.869482)		
			G	282 (0.530)	199 (0.614)				
			AA	51 (0.192)	19 (0.117)	0.037656		0.056796	0.090104
			AG	148 (0.556)	87 (0.537)				
			GG	67 (0.252)	56 (0.346)				
PADI2	rs2076616	17412501	A	239 (0.453)	124 (0.383)	0.045123	1.333854 (1.006036∼1.768493)		
			G	289 (0.547)	200 (0.617)				
			AA	56 (0.212)	19 (0.117)				
			AG	127 (0.481)	86 (0.531)	0.043905		0.635518	0.115895
			GG	81 (0.307)	57 (0.352)				
PADI2	rs10788656	17437330	C	66 (0.124)	33 (0.101)	0.309632	1.257511 (0.807708∼1.957803)		
			G	466 (0.876)	293 (0.899)				
			CC		1 (0.006)	0.57805		0.537095	0.563949
			CG	60 (0.226)	31 (0.190)				
			GG	203 (0.763)	131 (0.804)				
PADI2	rs1005753	17444769	A	469 (0.882)	294 (0.902)	0.358648	0.810280 [0.516780∼1.270469]		
			C	63 (0.118)	32 (0.098)				
			AA	206 (0.774)	132 (0.810)	0.636718		0.66802	0.613705
			AC	57 (0.214)	30 (0.184)				
			CC	3 (0.011)	1 (0.006)				
PADI1	rs2977310	17564990	A	276 (0.527)	191 (0.586)	0.091869	0.786607 (0.594953∼1.039999)		
			G	248 (0.473)	135 (0.414)				
			AA	72 (0.275)	60 (0.368)	0.129296		0.86481	0.191385
			AG	132 (0.504)	71 (0.436)				
			GG	58 (0.221)	32 (0.196)				
PADI4	rs1886301	17635411	A	339 (0.737)	226 (0.779)	0.190134	0.793388 (0.561018∼1.122006)		
			G	121 (0.263)	64 (0.221)				
			AA	128 (0.557)	86 (0.593)	0.177443		0.29393	0.319446
			AG	83 (0.361)	54 (0.372)				
			GG	19 (0.083)	5 (0.034)				
PADI4	rs11203368	17666508	A	265 (0.506)	207 (0.635)	0.000229	0.588197 (0.443155∼0.780711)		
			G	259 (0.494)	119 (0.365)				
			AA	69 (0.263)	62 (0.380)	0.00063		0.622611	0.208862
			AG	127 (0.485)	83 (0.509)				
			GG	66 (0.252)	18 (0.110)				
PADI4	rs6683201	17681277	A	115 (0.216)	61 (0.187)	0.306454	1.198058 [0.847182∼1.694256]		
			G	417 (0.784)	265 (0.813)				
			AA	10 (0.038)	5 (0.031)	0.564468		0.379357	0.7158
			AG	95 (0.357)	51 (0.313)				
			GG	161 (0.605)	107 (0.656)				
PADI4	rs1635564	17683526	A	110 (0.207)	51 (0.156)	0.066924	1.405539 (0.975591∼2.024967)		
			C	422 (0.793)	275 (0.844)				
			AA	11 (0.041)	5 (0.031)	0.160672		0.88935	0.548602
			AC	88 (0.331)	41 (0.252)				
			CC	167 (0.628)	117 (0.718)				
PADI4	rs1635561	17687027	A	158 (0.303)	102 (0.313)	0.753974	0.953243 (0.706556∼1.286058)		
			G	364 (0.697)	224 (0.687)				
			AA	25 (0.096)	15 (0.092)	0.851674		0.749657	0.727342
			AG	108 (0.414)	72 (0.442)				
			GG	128 (0.490)	76 (0.466)				
PADI6	rs12563231	17703861	A	145 (0.277)	94 (0.290)	0.673318	0.936114 (0.688707∼1.272398)		
			G	379 (0.723)	230 (0.710)				
			AA	23 (0.088)	15 (0.093)	0.907106		0.36451	0.602794
			AG	99 (0.378)	64 (0.395)				
			GG	140 (0.534)	83 (0.512)				
PADI6	rs1204898	17707739	A	77 (0.145)	54 (0.166)	0.408602	0.852422 (0.583554∼1.245167)		
			G	455 (0.855)	272 (0.834)				
			AA	3 (0.011)	7 (0.043)	0.103254		0.202677	0.152069
			AG	71 (0.267)	40 (0.245)				
			GG	192 (0.722)	116 (0.712)				
PADI6	rs10788668	17707798	A	303 (0.583)	177 (0.543)	0.256157	1.175428 [0.889214∼1.553765]		
			G	217 (0.417)	149 (0.457)				
			AA	85 (0.327)	44 (0.270)	0.4512		0.403189	0.201131
			AG	133 (0.512)	89 (0.546)				
			GG	42 (0.162)	30 (0.184)				
PADI6	rs2526839	17717312	A	353 (0.664)	214 (0.656)	0.831349	1.032110 (0.771632∼1.380517)		
			C	179 (0.336)	112 (0.344)				
			AA	114 (0.429)	68 (0.417)	0.973164		0.392476	0.43678
			AC	125 (0.470)	78 (0.479)				
			CC	27 (0.102)	17 (0.104)				
PADI6	rs6695849	17720439	A	304 (0.571)	174 (0.534)	0.28079	1.164751 (0.882771∼1.536802)		
			G	228 (0.429)	152 (0.466)				
			AA	70 (0.263)	38 (0.233)	0.384543		2.48E-05	0.007956
			AG	164 (0.617)	98 (0.601)				
			GG	32 (0.120)	27 (0.166)				
PADI6	rs7538876	17722363	A	90 (0.169)	50 (0.153)	0.543308	1.123982 [0.770960∼1.638653]		
			G	442 (0.831)	276 (0.847)				
			AA	5 (0.019)	4 (0.025)	0.600101		0.254433	0.920414
			AG	80 (0.301)	42 (0.258)				
			GG	181 (0.680)	117 (0.718)				

**Table 2:** Allele and genotype frequencies in a case control (n = 163) cohort of patients with AS (n = 51) by the illumina microassay.

Gene	dbSNP identity	Allele/Genotype	No. of patients with RA (%)	No. of controls (%)	Fisher’s p value	Odds Ratio (%95 CI)	Case HWE (df = 1)	Cotrol HWE (df = 1)
PADI1	rs2977310	A	53 (0.520)	191 (0.586)	0.23801	0.764505 (0.489129∼1.194914)		
		G	49 (0.480)	135 (0.414)				
		AA	14 (0.275)	60 (0.368)	0.46557		0.89717	0.191385
		AG	25 (0.490)	71 (0.436)				
		GG	12 (0.235)	32 (0.196)				
PADI2	rs2235926	A	39 (0.476)	125 (0.406)	0.25546	1.327814 (0.813896∼2.166234)		
		G	43 (0.524)	183 (0.594)				
		AA	4 (0.098)	20 (0.130)	0.05157		0.000964	0.07304
		AG	31 (0.756)	85 (0.552)				
		GG	6 (0.146)	49 (0.318)				
PADI2	rs2057094	A	40 (0.392)	125 (0.386)	0.90854	1.027097 (0.650915∼1.620685)		
		G	62 (0.608)	199 (0.614)				
		AA	4 (0.078)	19 (0.117)	0.4919		0.024013	0.090043
		AG	32 (0.627)	87 (0.537)				
		GG	15 (0.294)	56 (0.346)				
PADI2	rs2076616	A	40 (0.417)	124 (0.383)	0.54928	1.152074 (0.724747∼1.831362)		
		G	56 (0.583)	200 (0.617)				
		AA	5 (0.104)	19 (0.117)	0.50189		0.047816	0.115825
		AG	30 (0.625)	86 (0.531)				
		GG	13 (0.271)	57 (0.352)				
PADI2	rs10788656	C	15 (0.147)	33 (0.101)	0.20053	1.530825 (0.794713∼2.948770)		
		G	87 (0.853)	293 (0.899)				
		CC	1 (0.020)	1 (0.006)	0.39693		0.908514	0.563946
		CG	13 (0.255)	31 (0.190)				
		GG	37 (0.725)	131 (0.804)				
PADI2	rs1005753	A	88 (0.863)	294 (0.902)	0.26593	0.684159 (0.349498∼1.339275)		
		C	14 (0.137)	32 (0.098)				
		AA	39 (0.765)	132 (0.810)	0.20593		0.219174	0.613706
		AC	10 (0.196)	30 (0.184)				
		CC	2 (0.039)	1 (0.006)				
PADI4	rs1886301	A	78 (0.812)	226 (0.779)	0.49079	1.227139 (0.685128∼2.197939)		
		G	18 (0.188)	64 (0.221)				
		AA	34 (0.708)	86 (0.593)	0.06265		0.028475	0.319385
		AG	10 (0.208)	54 (0.372)				
		GG	4 (0.083)	5 (0.034)				
PADI4	rs11203368	A	63 (0.630)	207 (0.635)	0.92811	0.978848 (0.615232∼1.557369)		
		G	37 (0.370)	119 (0.365)				
		AA	18 (0.360)	62 (0.380)	0.92724		0.262968	0.208783
		AG	27 (0.540)	83 (0.509)				
		GG	5 (0.100)	18 (0.110)				
PADI4	rs6683201	A	29 (0.284)	61 (0.187)	0.03557	1.725803 (1.033839∼2.880908)		
		G	73 (0.716)	265 (0.813)				
		AA	3 (0.059)	5 (0.031)	0.09518		0.439825	0.715797
		AG	23 (0.451)	51 (0.313)				
		GG	25 (0.490)	107 (0.656)				
PADI4	rs1635564	A	28 (0.275)	51 (0.156)	0.00733	2.040276 (1.203749∼3.458134)		
		C	74 (0.725)	275 (0.844)				
		AA	5 (0.098)	5 (0.031)	0.03256		0.415989	0.548596
		AC	18 (0.353)	41 (0.252)				
		CC	28 (0.549)	117 (0.718)				
PADI4	rs1635561	A	35 (0.350)	102 (0.313)	0.487	1.182504 (0.736896∼1.897574)		
		G	65 (0.650)	224 (0.687)				
		AA	7 (0.140)	15 (0.092)	0.6215		0.586515	0.727339
		AG	21 (0.420)	72 (0.442)				
		GG	22 (0.440)	76 (0.466)				
PADI6	rs12563231	A	29 (0.290)	94 (0.290)	0.9981	0.999401 (0.609747∼1.638060)		
		G	71 (0.710)	230 (0.710)				
		AA	3 (0.060)	15 (0.093)	0.62196		0.407909	0.602794
		AG	23 (0.460)	64 (0.395)				
		GG	24 (0.480)	83 (0.512)				
PADI6	rs1204898	A	7 (0.070)	54 (0.166)	0.01695	0.379132 (0.166680∼0.862378)		
		G	93 (0.930)	272 (0.834)				
		AA	0 (0.000)	7 (0.043)	0.07432		0.594589	0.151992
		AG	7 (0.140)	40 (0.245)				
		GG	43 (0.860)	116 (0.712)				
PADI6	rs10788668	A	67 (0.657)	177 (0.543)	0.04259	1.611461 (1.014006∼2.560936)		
		G	35 (0.343)	149 (0.457)				
		AA	19 (0.373)	44 (0.270)	0.06813		0.061938	0.201051
		AG	29 (0.569)	89 (0.546)				
		GG	3 (0.059)	30 (0.184)				
PADI6	rs2526839	A	64 (0.627)	214 (0.656)	0.59228	0.881456 (0.555381∼1.398976)		
		C	38 (0.373)	112 (0.344)				
		AA	15 (0.294)	68 (0.417)	0.04995		0.002358	0.43675
		AC	34 (0.667)	78 (0.479)				
		CC	2 (0.039)	17 (0.104)				
PADI6	rs6695849	A	48 (0.471)	174 (0.534)	0.26529	0.776501 (0.497321∼1.212403)		
		G	54 (0.529)	152 (0.466)				
		AA	4 (0.078)	38 (0.233)	0.03099		4.18E-05	0.007948
		AG	40 (0.784)	98 (0.601)				
		GG	7 (0.137)	27 (0.166)				
PADI6	rs7538876	A	19 (0.186)	50 (0.153)	0.43038	1.263614 (0.705779∼2.262355)		
		G	83 (0.814)	276 (0.847)				
		AA	3 (0.059)	4 (0.025)	0.48406		0.255665	0.920414
		AG	13 (0.255)	42 (0.258)				
		GG	35 (0.686)	117 (0.718)				

**Figure 1:**
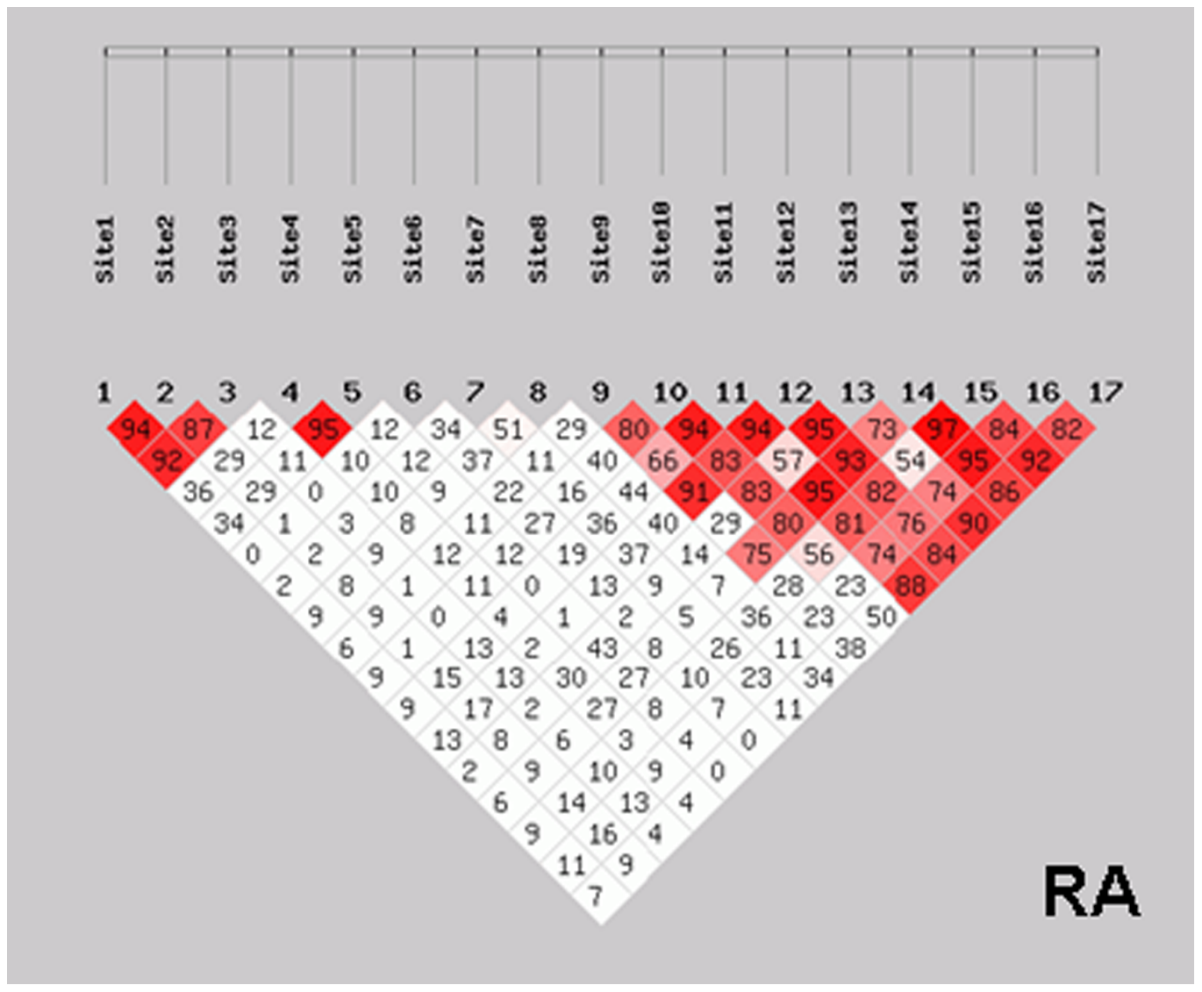
A linkage disequilibrium plot of the genotyped SNPs in the PAD region of in the RA group. Red areas represent higher levels of LD. Dark triangles represent haplotype blocks. Site 1–17 repsent rs2235926, rs2057094, rs2076616, rs10788656, rs1005753, rs2977310, rs1886301, rs11203368, rs6683201, rs1635564, rs1635561, rs12563231, rs1204898, rs10788668, rs2526839, rs6695849 and rs7538876, respectively.

To evaluate the extent of linkage disequilibrium (LD), the D′ was calculated between all possible pairs of polymorphisms. An LD analysis defined two blocks in the PAD locus in the RA population. One block was comprised of rs2235926, rs2057094 and rs2076616, with a mean pair-wise D′ of 0.923. Another block was comprised of rs6683201, rs1635564, rs1635561, rs12563231, rs1204898, rs10788668, rs2526839, rs6695849 and rs7538876 with a mean pair-wise D′ of 0.923. The results are presented in **Figure 1**.

An additional genotyping experiment was performed in a larger cohort of RA patients and AS patients using the Sequenom MassARRAY. The samples used in this study were independent of those used in the above microarray. The allele and genotype frequencies of all five SNPs did not deviate from Hardy-Weinberg equilibrium in the cases or controls. The allele and genotype frequencies for SNP rs2235926 [p = 0.000839 and 0.005134, respectively; Odds Ratio (95% CI) = 1.706733 (1.576366–1.866587) ] had a statistically significant association with RA. The allele and genotype frequencies for SNP rs2076616 [p = 0.003291 and 0.015558, respectively; Odds Ratio (95% CI) = 1.360432 (1.107805–1.670670) ] also showed a significant association with RA. The other three SNPs were not significantly associated with RA. No SNP had a statistically significant association with AS. The results are shown in **Table 3**. Following multiple-test correction, these SNPS still showed a significant difference in allelic frequency and genotypic frequency between the RA group and the controls. This result is in accordance with the results of the Illumina microarray.

**Table 3:** Allele and genotype frequencies in a case control cohort of RA patients (n = 307), AS patients (n = 324) and the health (n = 509) by sequenom.

Gene/Diseases	dbSNP identity	Allele/Genotype	No. of patients with RA (%)	No. of controls (%)	Fisher’s p value	Odds Ratio (%95 CI)	Case HWE (df = 1)	Control HWE (df = 1)
rs2235926	PADI2	A	240 (0.392)	482 (0.477)	8E-04	1.706733 (1.576366∼1.866587)		
RA		G	372 (0.608)	528 (0.523)				
		AA	50 (0.163)	123 (0.244)	0.005		0.481	0.154
		AG	140 (0.458)	236 (0.467)				
		GG	116 (0.379)	146 (0.289)				
rs2235926	PADI2	A	303 (0.475)	482 (0.477)	0.927	0.990797 (0.812435∼1.208317)		
AS		G	335 (0.525)	528 (0.523)				
		AA	77 (0.241)	123 (0.244)	0.996		0.257	0.154
		AG	149 (0.467)	236 (0.467)				
		GG	93 (0.292)	146 (0.289)				
rs2076616	PADI2	A	228 (0.371)	450 (0.446)	0.003	1.360432 (1.107805∼1.670670)		
RA		G	386 (0.629)	560 (0.554)				
		AA	44 (0.143)	106 (0.210)	0.016		0.683	0.3
		AG	140 (0.456)	238 (0.471)				
		GG	123 (0.401)	161 (0.319)				
rs2076616	PADI2	A	300 (0.466)	450 (0.446)	0.419	0.921429 (0.755596∼1.123656)		
AS		G	344 (0.534)	560 (0.554)				
		AA	76 (0.236)	106 (0.210)	0.671		0.17	0.3
		AG	148 (0.460)	238 (0.471)				
		GG	98 (0.304)	161 (0.319)				

We investigated the possible association between RF, anti-CCP antibody levels and the DNA polymorphisms of the 17 SNPs. No significant association was obtained between these PAD variants and RF or anti-CCP levels in patients with RA.

### PADI2 Expression in Synovial Tissue from RA, AS and OA Patients

**Figure 2:**
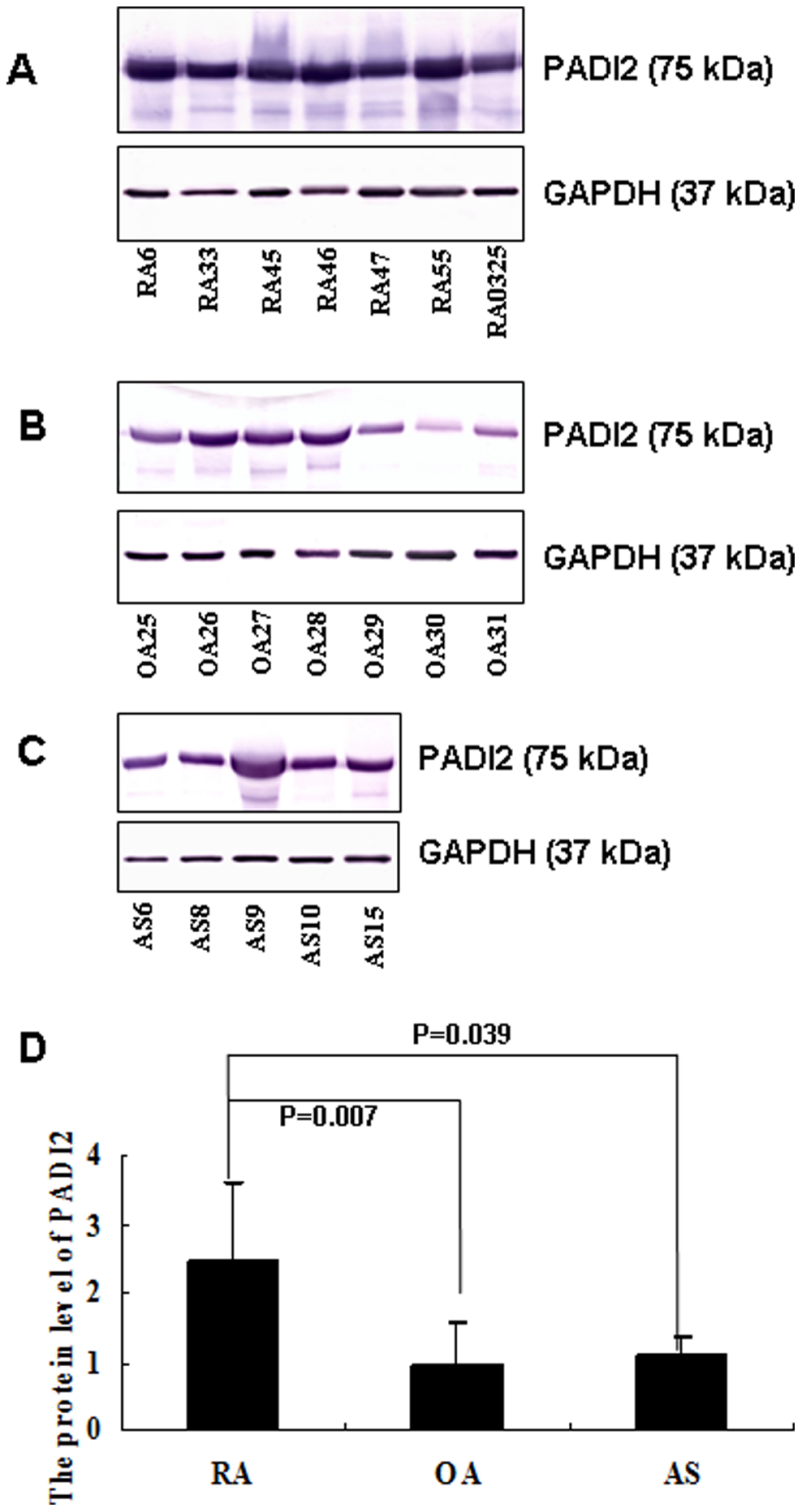
Semi-quantitative western blot analysis of PADI2 proteins in RA (A), OA (B) and AS (C) synovial tissues. The loaded tissue extracts were probed with an antibody to GAPDH to normalize the loading volume (D).

A western blot analysis was used to evaluate PADI2 expression in synovial tissues from RA, OA and AS patients. The expression levels were determined by normalizing their expression to GAPDH (37 kDa). The expression of PADI2 (75 kDa) was significantly increased in samples from RA patients compared to samples from patients with OA (p = 0.007) and AS (p = 0.039). The expression of PADI2 was not significantly different in samples from patients with AS compared to samples from patients with OA. The results are shown in **Figure 2**.

## Discussion

In our study, we used a custom-designed Illumina microarray to genotype 17 tag SNPs in the PAD locus to determine their association with susceptibility to RA. These 17 SNPs span the entire PAD region, including PADI1, PADI2, PADI3, PADI4 and PADI6. SNPs including rs2235926, rs2057094 and rs2076616 in the PADI2 locus showed a significant association with RA. We also detected a significant association between rs11203368 and RA. Because rs11203368 is located in the PADI4-encoding gene, our findings not only support previous finding regarding the association between PADI4 and RA susceptibility but also confirm the reliability of our genotyping results. The experiment demonstrated that other members of the PAD gene family are not associated with RA. To verify the above analysis, the Sequenom MassARRAY was used with a larger and independent cohort of RA patients. The study provided evidence that the SNPs rs2235926 and rs2076616 are significantly associated with RA. The Sequenom MassARRAY did not detect a significant association between SNPs in the PAD locus and AS, indicating a particular association between PADI2 and RA. Thus, both the microarray and Sequenom analyses provide evidence that PADI2 is associated with the risk of developing RA in the Northern Chinese population.

An LD analysis defined two blocks in the PAD locus among the RA population. One block was comprised of rs2235926, rs2057094 and rs2076616 in the PADI2 gene with a mean pair-wise D′ of 0.923; the other block was comprised of all other SNPs in PADI4 and PADI6. The results indicate that the strong association between PADI2 and RA is not a reflection of the association between PADI4 and the disease.

Too *et* *al.* genotyped 320 SNPs from the PADI locus (including PADI1, PADI2, PADI3, PADI4 and PADI6 genes) in 1,238 RA cases and 1,571 control subjects from Malaysia in a case-control study. They also conducted a meta-analysis including previously published RA data from the East Asian population. A haplotype analysis revealed four PADI4 SNPs with a significant association with RA. In addition, they detected a novel association between the PADI2 genetic variant rs1005753 and RA [34]. Lee *et* *al.* conducted a meta-analysis using 4,429 SNPs in 1,527 cases of RA and 3,421 controls in a Korean RA GWAS dataset. An ICSN Pathway analysis (identifying candidate causal SNPs and pathways) identified three candidates of causal non-HLA SNPs and four candidates of causal pathways involving the PADI4, MTR, PADI2 and TPH2 genes in RA [35]. Although our results showed that rs1005753 was not associated with RA, the genotyping data demonstrated that other PADI2 genetic variants have a significant association with RA, which is in accordance with the results of the above two meta-analyses. Our results and the meta-analyses performed by others suggest that polymorphisms in the PADI2 gene are associated with risk of RA in the Asian population.

Many studies have investigated the pathogenic role and regulatory mechanism of PADI2 in RA. Arandjelovic *et* *al.* identified PADI2 expression in mast cells from RA synovial fluid. They also found that activation of the P2X7 purinergic receptor (P2X7) induces PADI2 activity and robust protein citrullination. P2X7-mediated activation of PADI2 is sensitive to p38 MAPK and protein kinase C inhibitors, and PADI2 regulates the expression of TNFR2, ADAMTS9 and Rab6b transcripts in mast cells [36]. Within RA synovial tissues, the activation of macrophages and fibroblasts mediated by T-cell contact or driven by cytokines plays a prominent part in RA pathogenesis. Ferrari-Lacraz *et* *al.* found that PADI2 and PADI4 mRNA and proteins were transiently up-regulated in monocytes after contact with T-lymphocytes. Stimulation with IL-1β or IFN-β did not modify levels of PADI2 and PADI4 mRNA, but did enhance PADI4 protein expression. No mRNA or protein of any PAD isotype was detected in resting or stimulated synovial fibroblasts. Thus, they suggested that contact between stimulated T cells and monocytes/macrophages or cytokine-activated monocytes−/macrophages constitutes a highly likely source of PADI2 and PADI4, which are observed in inflamed synovial tissues [37]. Calabrese *et* *al.* conducted a methylation pattern analysis and detected a CpG island in the PADI2 promoter. They showed that the over-expression of PADI2 was associated with promoter demethylation [38]. Lee *et* *al.* examined PAD-mediated citrullination and its effect on pro-inflammatory activity. They found that PADI2 overexpression reduced NF-κB activity [39]. Our study detected increased PADI2 expression in synovial tissues from RA patients compared with samples from OA patients and AS patients. These previous studies suggest that PADI2 and its citrullination play an important role in RA autoimmunity. Nakayama-Hamada *et* *al.* suggested that PADI2 and PADI4 have different roles in different physiological and pathological conditions [40].

Bodnár *et* *al.* detected high levels of anti-mutated citrullinated vimentin (anti-MCV) in the serum of AS patients [41]. Bay-Jensen *et* *al.* detected high levels of matrix metalloproteinase-degraded fragments of vimentin in the serum of AS patients [42]. Their studies suggest that citrullination may be relevant in the pathogenesis of AS. Chen *et* *al.* investigated the PADI4 polymorphisms in AS in the Chinese Han population. Their study was conducted in a total of 316 Chinese patients of Han nationality with AS and 439 healthy controls. They genotyped 5 SNPs including rs11203366, rs11203367, rs874881, rs2240340 and rs1748033 in the PADI4 gene. No significant differences with regards to the frequency of alleles and genotypes were found when comparing the cases and controls [43]. The author thus suggested that PADI4 polymorphisms may not play an important role in the development of AS in the Chinese Han population. In this study, we found that rs1635564 in PADI4 is significantly associated with RA. We previously detected the expression of PADI4 in synovial membranes and synovial fluid of AS patients, although the levels were lower than those in tissues from patients with RA [33]. The involvement of PADI4 in the pathogenesis of AS warrants further study.

In summary, our study detected a significant association between polymorphisms in the PADI2 gene and RA. We also detected increased PADI2 expression in RA synovial tissues. Further studies are needed to better understand the role of PADI2 in the initiation and progression of RA.

## Materials and Methods

### Sample Preparation

Peripheral blood samples were collected from patients with RA or AS. RA was diagnosed according to the criteria of the American College of Rheumatology. RA patients had high levels of C-reactive protein (30–100 mg/liter, mean 24 mg/liter), anti-CCP (300–3,000 U/ml) and rheumatoid factor (RF) (160–2,560 U/ml). The diagnosis of AS patients was consistent with the modified New York criteria for AS. The patients with AS were positive for the HLA-B27 antigen. The healthy individuals were blood donors without a personal or family history of serious illness. Patients and healthy controls were selected from the same population living in the Shandong area of Northern China. The blood samples were collected in Monovette tubes containing 3.8% sodium citrate.

Synovial tissue samples were collected during knee joint replacement surgery in patients with RA (n = 7; 4 females; range, 33–68 years old; mean, 53 years old). RA patients had disease durations of 3–9 years and were classified as having erosive RA (Larsen class IV–V). Synovial tissues were also collected during joint replacement surgery for patients with OA (n = 7; 3 females; age range 40–72 years old; mean, 60 years old). The participants symptoms fulfilled the modified New York criteria. Synovial tissue samples from patients with AS (n = 5, 2 female, 28–54 years old, mean 35) were collected during hip joint replacement surgery. AS patients had an average disease duration of seven years and were positive for HLA-B27 antigen. Patients with AS and RA took disease-modifying antirheumatic drugs (DMARDs) before surgery. Patients with AS, RA and OA were also medicated with non-steroidal anti-inflammatory drugs (NSAIDs). Thus, the medical pretreatment did not influence the results, and the experimental results are comparable between groups. Synovial samples were dissected from the connective tissues and immediately stored at −80°C for later use.

Both patients and healthy controls gave their written consent to participate in the study and allowed their biological samples to be genetically analyzed. The Ethical Committee of Shandong Provincial Qianfoshan Hospital approved this study.

### Genomic DNA Isolation, SNP Selection and Genotyping

Genomic DNA was extracted from whole blood samples using the Omega E-Z 96 Blood DNA kit (Omega, USA) according to the manufacturer’s protocol. After extraction, the genomic DNA was diluted to a final concentration of 15–20 ng/µl for use in the genotyping assays.

Tag single nucleotide polymorphisms (tag SNPs) across the PAD locus were identified by searching the HapMap database [44], [45]. Only SNPs with a minor allele frequency (MAF) greater than 5% and a pair-wise r^2^≥0.8 were considered. Candidate SNPs were submitted to Illumina for a design score. Finally, seventeen SNPs were selected, as described in **Table 1**.

The seventeen SNPs in **Table 1** were genotyped using a custom-designed Illumina 96-SNP VeraCode microarray. Peripheral blood samples were collected from patients with RA (n = 267, 183 females) and AS (n = 51, 10 females). RA patients had a mean age of 51.7 years, while AS patients had a mean age of 35.9 years. A total of 160 (58 females) healthy individuals with a mean age of 48.0 years were blood donors. The work was completed at Beijing Yimei Tongde, a China-based company, which provided technical services for the genotyping.

The microarray results were verified with an allele-specific MALDI-TOF mass spectrometry assay (Sequenom MassARRAY). Five SNPs, including rs2235926, rs2057094, rs2076616, rs1204898 and rs10788668, were genotyped in a cohort of 307 patients with RA (252 females), 324 patients with AS (45 females) and 509 healthy controls (194 females), none of whom were included in the experiments utilizing the Illumina microarray. The RA patients had a mean age of 51.43 years, the AS patients had a mean age of 28.88 years, and the healthy individuals had a mean age of 62.88 years. The polymorphism-spanning fragments were amplified by the polymerase chain reaction (PCR), and genotyping was performed using the Sequenom MassARRAY iPLEX platform. Primers for the amplification and extension reactions were designed using MassARRAY Assay Design version 3.1 software (Sequenom, San Diego, CA). The SNP genotypes were obtained according to the protocol provided by the manufacturer. The work was completed at Bioyoung Tech, a China-based company, which also provided technical services for the genotyping.

The genotyping quality was evaluated by a detailed quality control procedure consisting of a >95% success identification rate, duplicate identification of the genotypes, internal positive control samples and testing for the Hardy-Weinberg Equilibrium (HWE). The SNPs were analyzed for association by comparing the MAF between the cases and controls. Dominant and recessive models were considered with respect to the minor allele. Associations between the SNPs and the diseases were evaluated using odds ratios (ORs) with 95% confidence intervals (CIs). Fisher’s exact test was used for comparisons between categorical variables. P values less than 0.05 were considered statistically significant. Genotypic associations were assessed using Plink v1.07 (http://pngu.mgh.harvard.edu/purcell/plink/) and SHEsis (http://analysis.bio-x.cn/myAnalysis.php) software [46]. A Bonferroni single-step correction was performed using the Plink v1.07. Linkage disequilibrium (LD), coefficient (D′ and r2) and haplotype was estimated by Haploview 4.2 (http://www.broad.mit.edu/mpg/haploview/) [47].

### Western Blot Analysis

A total of 200 µg of synovial tissue from the RA patients, OA patients and AS patients were homogenized in Cell Lysis Solution (Sigma) and centrifuged at 16000×g for 5 min at 4°C. The total protein was separated by sodium dodecyl sulfate–polyacrylamide gel electrophoresis (SDS-PAGE) and transferred onto nitrocellulose membranes (Amersham, USA). A Western blot analysis was conducted using antibodies against human PADI2 at a 2,000-fold dilution, run overnight at 4°C. The antibody (Abcam) was prepared in rabbits using a synthetic peptide conjugated to KLH derived from residues 100–200 of human PADI2. The immuno-signals were visualized using the Protein Detector BCIP/NBT Western Blot kit (Beyotime) following the manufacturer’s instructions. A separate membrane prepared by the same protocol was probed with an anti-GAPDH antibody (Santa Cruz, USA) to normalize the sample loading.
